# Macrophage-Based Therapies for Atherosclerosis Management

**DOI:** 10.1155/2020/8131754

**Published:** 2020-01-29

**Authors:** Renyi Peng, Hao Ji, Libo Jin, Sue Lin, Yijiang Huang, Ke Xu, Qinsi Yang, Da Sun, Wei Wu

**Affiliations:** ^1^Institute of Life Sciences, Wenzhou University, Wenzhou, Zhejiang 325035, China; ^2^Biomedical Collaborative Innovation Center of Zhejiang Province & Engineering Laboratory of Zhejiang Province for Pharmaceutical Development of Growth Factors, Biomedical Collaborative Innovation Center of Wenzhou, Wenzhou, Zhejiang 325035, China; ^3^Department of Orthopaedics, The Second Affiliated Hospital and Yuying Children's Hospital of Wenzhou Medical University, Wenzhou, Zhejiang 325027, China; ^4^Wenzhou Institute, University of Chinese Academy of Sciences, Wenzhou, Zhejiang 325000, China; ^5^Key Laboratory for Biorheological Science and Technology of Ministry of Education, State and Local Joint Engineering Laboratory for Vascular Implants, Bioengineering College of Chongqing University, Chongqing 400030, China

## Abstract

Atherosclerosis (AS), a typical chronic inflammatory vascular disease, is the main pathological basis of ischemic cardio/cerebrovascular disease (CVD). Long-term administration was characterized with low efficacy and serious side effects, while the macrophages with attractive intrinsic homing target have great potential in the efficient and safe management of AS. In this review, we focused on the systematical summary of the macrophage-based therapies in AS management, including macrophage autophagy, polarization, targeted delivery, microenvironment-triggered drug release, and macrophage- or macrophage membrane-based drug carrier. In conclusion, macrophage-based therapies have great promise to effectively manage AS in future research and clinic translation.

## 1. Introduction

**Figure 1:**
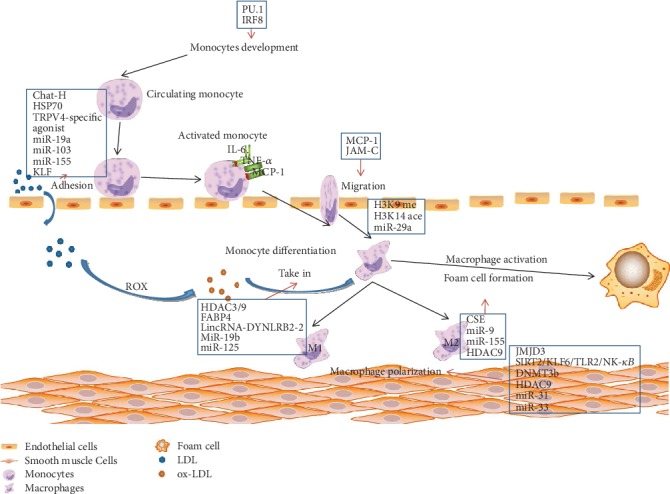
Illustration of monocyte and macrophage in AS. After recruited to endothelial cells, the active monocytes oversecrete IL-6, MCP-1, and TNF-*α* and subsequently differentiate into macrophages. Macrophages are polarized into two types: M1 and M2. Once macrophages uptake the ox-LDL and cholesterol, foam cells are formed and induced atherosclerotic progression. Reproduced with permission from [3], copyright 2017 Wiley.

Cardio/cerebrovascular disease (CVD) is the leading cause of morbidity and mortality worldwide. Atherosclerosis (AS) is considered the main pathological basis of ischemic CVD, including cerebrovascular disease, coronary heart disease, and peripheral arterial disease. AS is a typical chronic inflammatory vascular disease due to the accumulation of a large amount of lipids in the arterial wall, especially in the branched and bended arteries [1, 2]. The representative macrophages play an important role in the pathological progression in lesions of AS (Figure 1) [3]. The migration, activation, infiltration, and proliferation of macrophages lead to inflammation-mediated atherosclerotic plaque formation [4]. Furthermore, macrophages secrete abundant proteases and tissue factors to promote inflammation, lipid deposition, and plaque rapture. Thus, macrophages are regarded as an attractive target for managing AS [5, 6]. Despite the wide clinical use of local anti-inflammatory drugs, the traditional therapies have low bioavailability and severe side effects, far from meeting the long-term dosing requirements for the significant AS management in safety and efficiency [7]. This review discussed recent research studies on the role of macrophages in the pathogenesis of AS, especially systematically highlighting the advanced strategies in macrophage-based therapies for AS management, such as macrophage autophagy, polarization, targeted delivery, microenvironment-triggered drug release, and macrophage- or macrophage membrane-based drug carrier.

## 2. Macrophages in AS

### 2.1. Macrophages in the Early Stage

In the early stage of AS, low-density lipoprotein (LDL) accumulates in the intima of blood vessels, activating the endothelium to express leukocyte adhesion molecules and chemokines and promoting the recruitment of monocytes and T cells [8, 9]. The macrophage colony-stimulating factor (M-CSF) and other differentiation factors accelerate differentiation of monocytes into macrophages, which upregulate pattern recognition receptors (PRRs), including toll-like receptors (TLRs) and scavenger receptors (SRs) [10]. The activation of the TLR pathway leads to an inflammatory response, while the SR pathway regulates the oxidized low-density lipoprotein (ox-LDL) resulting in foam cell formation. During the early stage of inflammation in the AS process, activated monocytes and lymphocytes absorb ox-LDL by SRs and promote foam cell transformation, and interaction with foam cell and accumulation of various factors contribute to the pathogenesis of atherosclerosis [11], while reduction in foam cell formation or ox-LDL uptake was verified to reduce the atherosclerotic plaque burden [12, 13]. ATP-binding cassette (ABC) transporters expressed by macrophages are involved in cholesterol reversal and reducing plasma cholesterol level [14]. ABCA1 and ABCG1 transporters reverse cholesterol transport and generate HDL, which affect the atherosclerotic progression. The genes encoding ABCA1 and ABCG1 are transcriptionally upregulated in response to the elevated cellular cholesterol levels [15], especially in the early stage. It had been shown that ABCA1 and ABCG1 gene knockout mice led to a large amount of lipid accumulation and foam cell formation in macrophages [16]. Furthermore, ABCA1 and ABCG1 are related to cell apoptosis and release of inflammatory factors. Studies showed that macrophages express high levels of ABCG1 and the multiple inflammatory genes in macrophages, which is consistent with the intracellular accumulation of multiple factors and promoted the progress of AS [16]. Deficiency of ABCA1 or ABCG1 in mice increased the apoptosis in macrophages and the inflammation in plaque, while apoptosis of macrophages in ABCA1- and ABCG1-deficient mice was increased, but the atherosclerotic progression was inhibited [17]. ABCA1 and ABCG1, as the cholesterol efflux transports, promote cholesterol efflux from cells by transporting phospholipids and cholesterol to high-density lipoprotein or free apolipoprotein A-I [18]. Therefore, ABCA1 deficiency and ABCG1 deficiency will cause inflammatory activation of macrophages leading to AS pathological deterioration [19].

### 2.2. Macrophages in the Progression Stage

Macrophages play an important role in promoting plaque formation, diluting fibrous cap and necrotic core components, which leads to the increased inflammatory response and apoptotic signals of smooth muscle cells (SMCs) and leukocytes in atherosclerotic plaques [20, 21]. Moreover, macrophages reduce the amount of intimal fibroblast-like SMCs and degrade the collagen by oversecreting matrix metalloproteinase (MMP). In the site of vascular injury, macrophage apoptosis and phagocytic clearance of apoptotic cells resulted in increased necrotic core and decreased plaque stability [8, 22]. The rupture site of plaque is almost always located near the necrotic core of plaque, which is related to the diluent fiber cap. Thus, the strength of the fiber cap is an important indicator to determine the stability of plaque for the potential risk evaluation of plaque rupture [23]. Mhem, a nonfoam protective macrophage, stabilized plaque by reducing foam cell formation and enhancing anti-inflammation and tissue regeneration at the advanced stage of AS [24]. Mox, a macrophage induced by phospholipid oxide, also has a potential to be used for AS protection [7]. Mox macrophages, rich in advanced mouse lesions, play an atheroprotective role because the low-density lipoprotein receptor–deficient mice would emerge with accelerated atherogenesis which results from myeloid deficiency of Nrf2 [25]. Additionally, some inflammatory genes have upregulated expression in Mox macrophages in keeping with macrophages which respond to oxidized phospholipids by upregulation of inflammatory gene expression in wild-type mice [26].

## 3. Macrophage-Based Therapy Strategies

### 3.1. Inducing Macrophage Autophagy

**Figure 2:**
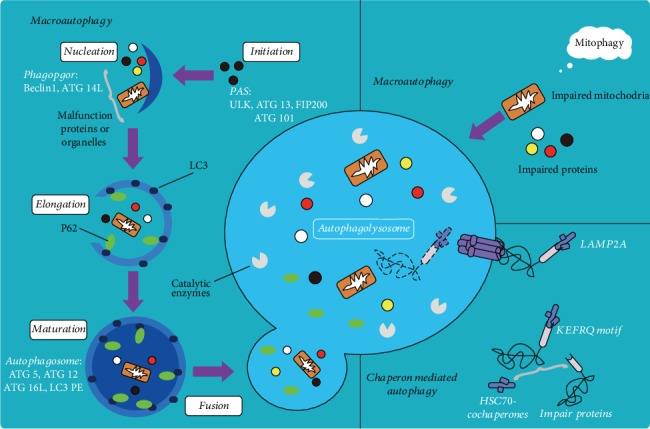
Illustration of macrophage autophagy in different routes. Reproduced with permission from [29], copyright 2019 Springer.

Autophagy is a major catabolic process, which functions in the maintenance of the cellular homeostasis in eukaryotic cells (Figure 2). It is activated under stress conditions such as nutrient deprivation, hypoxia, oxidative stress, and DNA damage [27, 28]. Further, autophagy may lead to the occurrence and development of various diseases, including malignant tumors, neurodegenerative diseases, cardiovascular diseases, and immune system disorders; thus, autophagy may be served as a potential strategy for the treatment of AS [27, 28].

Autophagy is a catabolic cellular process that degrades misfolded and dysfunctional proteins, cytoplasmic components, and organelles [28]. The formation of atherosclerotic plaque is enhanced in the mice lacking the key autophagy-related gene5 (ATG5). The abundant increase of autophagy chaperone p62 is a nonautophagic protective response to AS, suggesting that the autophagy of macrophages and its related regulators play an important role in AS [30]. During the whole process of autophagy, many genes related to autophagy are involved in regulation [31, 32]. mTOR is a highly conserved serine/threonine protein kinase that acts as a growth factor, a central sensor of cellular nutrition and energy state, and the core of autophagy regulation. mTOR exists in two different complexes, TORC1 and TORC2, which can integrate multiple signals from upstream pathways, inhibit ATG1(ULK1), and block the formation of autophagosomes [33]. mTORC1 inhibits autophagy by integrating upstream signals through the class I PI3K-PKB (also known as Akt) pathway when adequate nutrients are present. Under other stimuli, such as starvation and inflammatory oxidation, the activated class III PI3K-Beclin1 complex and the inactivated Beclin1/Bcl-2 complex are capable of inducing autophagy by promoting the assembly of ATG12-ATG5-ATG16L complex and ATG8/LC3. AMP-activated protein kinase (AMPK) inhibits mTORC1 activity and acts as a positive regulator of autophagy [32, 34]. Basal autophagy protects cells from environmental stimuli, while excessive autophagy leads to cell death and plaque instability, which is important for controlling the progression of AS. Inhibition of autophagy by silencing ATG5 or other autophagy mediators enhances the reductive coenzyme II oxidase-mediated oxidative stress and promotes the plaque necrosis and the deterioration of foam cells [35].

Nod-like receptor protein (NLRP3) inflammasome is closely related to the autophagy of AS and can be activated by the cholesterol crystals in plaques to promote the secretion of IL-1*β*, thus accelerating the development of AS [36]. Further, autophagy plays various roles in negatively regulating the activation of inflammasome, such as removing inflammasome-activating endogenous signals and isolating and degrading inflammasome components [37]. Autophagy removes the damaged mitochondria, reduces reactive oxygen species (ROS) production under stress, inhibits the activation of NLRP3, and even regulates NLRP3 activity by capturing and degrading the assembly of inflammasome complexes through the corresponding ubiquitination [38]. Furthermore, autophagy-induced mTOR inhibitors or AMPK activators reduce the inflammatory response to inhibit the development of AS plaques [39]. Shi et al. found that macrophage autophagy was enhanced and IL-1*β* secretion was reduced upon the starvation and mTOR inhibitor rapamycin treatment [40]. The PI3K/Akt/mTOR signaling pathway was blocked, while autophagy in macrophages was activated upon the rapamycin and mTOR-siRNA treatment in the rabbit model, which significantly inhibited the inflammatory response and enhanced the stability of plaques [41].

Autophagy inducers have been identified as an efficient and promising treatment for AS. Everolimus, a derivative of rapamycin and an inhibitor of mTOR, showed a significant therapeutic effect on malignancies including breast cancer and renal cell carcinoma [42]. Hsu et al. reported that the mRNA levels of autophagy were significantly increased and secreted protein expression was decreased by everolimus [43]. The results suggested that everolimus has great potential to inhibit AS by diminishing viability of foam cells, decreasing matrix degradation, and reducing the secretion of proinflammatory cytokine [43]. Resveratrol, a plant-derived polyphenolic compound, induces autophagy by inhibiting mTOR [42], which plays an important role in anti-AS and vasodilation [44]. Liu et al. reported that resveratrol could promote its cellular burial effect on ox-LDL-induced apoptotic RAW264.7 cells by activating Sirt1-mediated autophagy [45].

MicroRNAs (miRNA) regulate the autophagy through posttranscriptional repression of autophagy-related gene or upstream effectors [46, 47]. Therefore, miRNA regulation using miRNA inhibitors might be a potential therapeutic strategy for AS. Ouimet et al. identified miR-33 as a key posttranscriptional regulator of genes involved in cholesterol and fatty acid homeostasis [46]. They found that miR-33 inhibited apoptotic cell clearance via an autophagy-dependent mechanism, and macrophages treated with anti-miR-33 increased the efferocytosis, lysosomal biogenesis, and degradation of apoptotic cells [46].

Catechins (EGCG) activate autophagy by activating macrophages PI3K III. The anti-AS effect of berberine may also involve the activation of the AMPK/mTOR signaling pathway to induce autophagy and inhibit macrophage inflammatory response [48]. Berberine-induced autophagy inhibits cell inflammation induced by ox-LDL. Simvastatin can enhance macrophage autophagy induced by ox-LDL and reduce lipid aggregation and AS formation [49]. Ursolic acid, as a natural pentacyclic triterpenoid carboxylic acid, has the anti-AS potential to effectively enhance autophagy of macrophages and promote the cholesterol outflow of macrophages [49]. The anti-inflammatory effect is also related to the inactivation of the Akt/mTOR pathway and the inhibition of the secretion of IL-1*β* induced by lipopolysaccharide. Therefore, induction of macrophage autophagy can be a potential therapeutic strategy for AS [50].

### 3.2. Inducing Macrophage Polarization

Macrophages after activation included two main phenotypes: M1 type and M2 type. Subtype differentiation and related dysfunction of macrophages are the key steps to determine plaque progression and stability. Although both the M1 and M2 subtypes appear at the sclerotic lesion site, they show an opposite effect [21, 51]. Previous studies demonstrated that miRNA-216a activates telomerase, induces M1-type differentiation and aging, and promotes macrophage lipid uptake capacity and foam cell formation, thus accelerating the progression of atherosclerotic plaques [52, 53]. M1 macrophages respond to TLR and interferon-*γ* signaling, which can be induced by pathogen-associated molecular complexes (PAMP), lipopolysaccharide (LPS), and lipoproteins [54]. This type of macrophage can secrete proinflammatory cytokines such as tumor necrosis factor (TNF-*α*), interleukin-1*β* (IL-1*β*), IL-12, and IL-23 and the chemokines CXCL9, CXCL10, and CXCL11. High levels of ROS and nitric oxide (NO) can also be induced by proinflammatory macrophages, which contribute to further development of inflammatory responses [55, 56]. M2 macrophages with anti-inflammatory properties respond to Th2 cytokines IL-4 and IL-13 and secrete anti-inflammatory factors (such as IL-1 and IL-10 receptor agonists) [57]. M1 macrophages are accumulated in progressive plaques, while M2 in degenerative plaques contributing to tissue repair and remodeling. M1- and M2-type macrophages, cells with proinflammatory and anti-inflammatory functions during the development of AS, were mutually transformed to manage the progress of plaque stability [22].

A key feature of macrophages is the high plasticity in response to various microenvironmental stimuli. Macrophages are actively involved in the immune response for engulfing pathogens and cell debris and secreting proinflammatory factors [58]. However, some macrophages such as M1 type and Mhem play a role in eliminating inflammatory responses and promoting tissue remodeling [59]. The lesion site of AS provides a specific microenvironment, enriched with the activated cells, the modified lipoproteins, the proinflammatory factors, and the apoptotic cells [60]. A large number of proinflammatory M1-type macrophages were found in AS lesions. In addition, the atherosclerotic progression is positively correlated with the increased M1 macrophages; cells with a high expression of proinflammatory markers are preferentially located in the friable shoulder of plaque and the adventitia [61]. After activation of M1 macrophages, expression of inducible nitric oxide synthase (iNOS), CD86, and major histocompatibility complex II (MHC II) was upregulated, and multiple proinflammatory factors including tumor necrosis factor-*α* (TNF-*α*), interleukin-1 (IL-1), interleukin-12 (IL-12), and proinflammatory medium NO were secreted [4, 62]. These proinflammatory factors can lead to endothelial injury, promote oxidative stress, enhance apoptosis, and accelerate the calcification rate of necrotic nucleus [63].

Anti-inflammatory M2-type macrophages were identified in the stable plaque areas with small possibility to form foam cells. Therefore, the plaque progression can be reflected by proinflammatory and anti-inflammatory macrophage subtypes [26]. Transcriptome-based network analysis is a powerful modern tool for studying macrophage activation and function, providing a set of data on specific genes involved in different stages of macrophage activation [64]. Macrophage activation was analyzed by examining the changes in macrophage gene transcription induced by 28 different stimuli or combinations of stimuli. The study identified 49 groups of genes with similar transcriptional induction that respond to various stimuli and regulate specific transcription factors that promote macrophage activation [65]. In conclusion, the response of macrophages in different individuals to various stimuli is largely influenced by genetic variation, especially in genomic regulatory elements that coordinate macrophage induction and activation.

In the stage of lesion initiation and progression, macrophage accumulates within the subendothelium or neointima [18]. The presence of apoB lipoproteins and expression of endothelial adhesion molecules contribute to these early macrophages which accumulate in susceptible regions of arteries. In advanced necrotic lesions, macrophage apoptosis is increased and partly induced by lipoproteins or oxidized phospholipids. In the advanced lesion, macrophages could not clear these apoptotic cells, which results in increasing inflammation and plaque necrosis because of release of inflammatory mediators from the residual, postapoptotic necrotic cells. Macrophage autophagy contributes to protecting against lesion necrosis [66]. Lesion regression can be achieved in the hyperlipidemic mice by reducing the aggressive lipid, and that in diabetic mice by normalizing the blood glucose. The regression and resolution in these mice is characterized by altering relative gene expression and reducing lesion macrophage content. However, the operative mechanism in human subjects is as yet to explore [67].

There are some therapies that could be used for the regulation of macrophage phenotype. Anti-inflammatory cytokine secreted by macrophages plays an important role in the occurrence and development of AS. Also, its expression level is closely related to the process of AS [68, 69]. Studies have found that endothelial injury and increased aortic stiffness directly accelerate the process of AS, while anti-TNF-*α* therapy can reduce aortic stiffness, reduce inflammation, and slow down the process of AS [15]. CD40 and CD40L are expressed in macrophages, ECs, VSMCs, and other cells associated with AS lesions, and the CD40/CD40L system may be a biomarker for clinical evaluation of AS stability [70]. Regulation of miRNA has become a research hotspot in recent years; because of the important role of miRNA in cardiovascular and cerebrovascular diseases, it can be expected that miRNA may become a promising biomarker for clinical diagnosis or even a drug therapeutic target [71]. Therefore, studying the role of macrophages and related biological macromolecules in the development of atherosclerosis is of great significance for the in-depth understanding of the etiology and pathogenesis of atherosclerosis, as well as for its diagnosis, treatment, and drug development.

### 3.3. Macrophages for Targeted Delivery

In addition to the stability of cellular structure, the surface glycoproteins of macrophages play a crucial role in homing function to achieve targeted delivery to AS lesions. Studies have shown that about 45.8% known proteins on the surface of macrophages are membrane proteins annotated by the gene ontology with different functions, such as CD11b, CD14, CD18, CD40, CD86, CD44, and CD16 [21, 72, 73]. For example, the interaction between CD40 and soluble CD40 ligands regulates the expression of cytokines, chemokines, adhesion molecules, and growth factors and promotes the inflammation and immune response, inducing the atherosclerotic plaque development and vulnerability. In addition, C-reactive protein promotes the expression of VCAM-1, ICAM-1, e-selectin, and monocyte chemoattractant protein-1 and promotes inflammation, which is an important inflammatory marker in atherosclerotic progression [4, 6, 21, 74].

These membrane surface proteins can be used for the design of targeted drug delivery systems to macrophages. Yu et al. designed a pH-responsive polymeric micelle with further mannose modification to achieve CD206 (mannose receptor)-targeted siRNA delivery [75]. The mannosylated nanoparticles improve the delivery of siRNA into primary macrophages by 4-fold relative to the delivery of a nontargeted version of the same carrier [75]. Scavenger receptors (SRs) expressed in the activated macrophages are considered to be the most promising target biomarkers for targeted drug delivery [76–78]. Lewis et al. reported that glycosylated micelles competitively block macrophages SRs of MSR1 and CD36 to reduce the assimilation and accumulation of ox-LDL [79]. Small-molecule folate-targeted conjugates were found to specifically bind to the folate receptor-expressing macrophages *in vitro* and selectively accumulate at sites of inflammation *in vivo* [80–82]. A PEG-coated, acetic anhydride-capped, folate-targeted poly(amidoamine) (PAMAM) dendrimer was designed to deliver more cargo than small-molecule conjugates [82].

High-density lipoprotein (HDL) is an important plasma lipoprotein in the lipid transport system, which possesses several antiatherogenic functions including reverse cholesterol transport (RCT) and anti-inflammatory, antioxidant, and vascular protective properties [83–85]. Zhao et al. designed an atorvastatin calcium- (AT-) loaded dextran sulfate- (DXS-) coated core-shell reconstituted HDL (rHDL) [86]. Through the high affinity between DXS and scavenge receptor class AI (SR-AI), it was developed for the targeted drug delivery to macrophages and displayed biofunction of inhibiting ox-LDL uptake and promoting cholesterol efflux [86].

### 3.4. Macrophages for Triggering Cargo Release

More than 20% of cells are composed of macrophages in the well-known atherosclerotic lesions. Excessive ROS released by the active macrophages leads to oxidative/antioxidative imbalance [87]. The ROS may affect the conversion of LDL into ox-LDL, promoting the SMC death and accelerating the AS process [9, 21, 87]. Therefore, the excessive ROS might be a desirable target for triggering theranostic release in AS. Kim et al. designed a macrophage-targeted theranostic nanoparticle by coupling a photosensitizer with chlorin e6 hyaluronic acid [88]. The obtained nanoparticle MacTNP can be activated and emit near-infrared fluorescence by ROS in macrophage cells, which has great potential in selective NIR fluorescence imaging [88].

### 3.5. Macrophage- or Macrophage Membrane-Based Carrier

Compared with freely drug administration, carrier-mediated targeted drug delivery has been successfully used for the systemic delivery of a variety of antiatherogenic drugs with an enhancement in therapeutic efficacy and a reduction in side effects. However, carrier clearance by the immune system before reaching the targeted lesion is one of the major obstacles to efficient drug delivery due to the rigorous delivery demands *in vivo* [89, 90]. Besides, the biocompatibility and safety of artificially synthesized nanoparticles are much lower than those of natural materials. Further, the intrinsic sophisticated biofunctions of natural substances are difficult to construct due to their exceptional complex structures. Cell membrane coating carriers or cells have emerged as a promising therapeutic platform to evade the undesirable clearance through the biomimetic camouflage [91, 92]. Thus, cell- or cell membrane-based drug carriers have unique advantages in target activity, which significantly improve the bioavailability and reduce the side effects [93, 94]. A variety of chemokines in AS can be specifically recognized by macrophage to realize targeted therapy, termed macrophage homing. Thus, the macrophage membrane has been used to build a biomimetic drug delivery system for targeting AS with a good potential for accurate treatment of lesions, which provides the possibility for the macrophage cell membrane-based drug carrier with AS homing functions [95, 96]. In order to combine the reconfigurability of nanoparticles with the natural functions of cells, a drug carrier has been creatively combined with macrophage or macrophage membrane to construct the hybrid biomimetic drug delivery system with the tailorable functions [93, 97, 98]. Besides, the biomimetic carrier has excellent biocompatibility and low immunogenicity.

**Figure 3:**
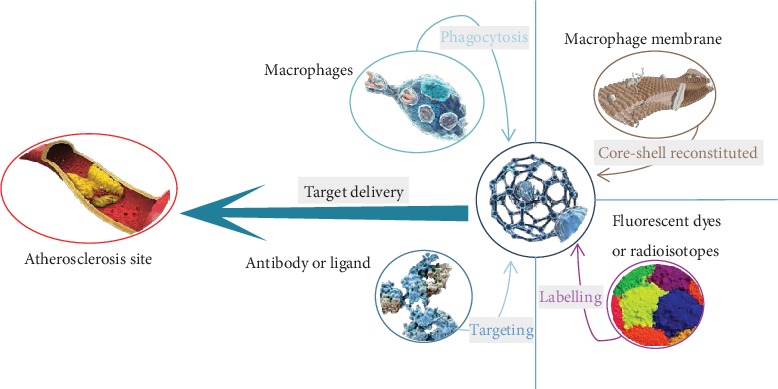
Illustration of functional modifications of the carrier.

In the macrophage drug carrier, the drug loads into macrophage mainly by *in vitro* incubation or *in vivo* direct injection. The *in vitro* incubation method is involved in adequately incubating macrophages with drugs *in vitro* and subsequently reinjecting the cargo-loaded macrophage carrier into the body for therapy. The *in vivo* direct injection method refers to a direct injection into the body using the modified specific ligands or drug delivery systems with appropriate particle size to harvest the phagocytic macrophages as therapeutic medicament [99, 100]. Development of the macrophage targeting can enhance the targeted cargo (diagnostic imaging and anti-inflammatory drugs) delivery to atherosclerotic lesions for diagnosis and therapy [101, 102]. Martinez et al. demonstrated the ability of rhodamine-labeled lipid films rehydrated with macrophage proteins targeting activated endothelia through CD11a and CD18, which both bind to ICAM-1 [103]. Due to the increased circulation time and homing capabilities, the platform may be loaded with therapeutics with diverse physical properties and hence show promise for use in magnetic resonance imaging applications. Nevertheless, the former method of directly loading drugs into macrophages has some deficiencies in preparation, such as low loading efficiency, premature drug release, and undesirable drug inactivation [61, 104, 105]. Therefore, the macrophage membrane-camouflaged drug carrier has been developed by using the macrophage membrane to further coat the drug carrier on the surface. Due to the surface modification of the macrophage membrane, the macrophage membrane carrier inherits the specific biological functions of the source cells, such as long circulation and AS-relevant homing. This design strategy lays the foundation for the development of the advanced cell membrane-based nanotherapeutics against AS. Based on the high affinity between *α*4*β*1 integrin and VCAM-1 in the macrophage membrane, Cheng and Li constructed a biomimetic “core-shell” structured nanoparticle with PLGA “core” and macrophage membrane “shell” to target drug delivery in AS lesions [106]. The results indicated that macrophage membrane-coated PLGA nanoparticle had a strong affinity to the target receptor VCAM-1, which could effectively identify the target cells and target tissues *in vivo*. With the variety of small molecules, biological macromolecules and tracer probe loading, polymeric and inorganic nanoparticles, liposomes, and abundant other bioactive carriers can be coated using the macrophage membrane to enhance the advanced theranostic application in AS (Figure 3).

## 4. Conclusion and Perspectives

Due to severe chronic inflammation, an extremely long-term administration has a high requirement for the efficacy and safety of AS treatment. The novel therapies based on the intrinsic cells are beneficial to reduce the undesirable clearance and immune responses. Macrophages play a critical role in the formation and progression of the atherosclerotic lesion. Artificially regulated or modified macrophages have been widely used, including regulating macrophage autophagy, inducing macrophage polarization, enhancing the active target delivery to macrophages, responding to the specific macrophage microenvironment for triggering drug release, and engineering the macrophage- or macrophage membrane-based drug carrier to efficiently manage AS lesion using the biomimetic therapy. In future studies, engineered macrophages with multifunctions will be further identified to self-adapt the atherosclerotic lesion for satisfying the advanced precision and personalized therapy. Therefore, macrophage-based therapy represents a novel platform with considerable potential for effective and safe AS management in future research and clinic translation applications.
